# Self-focused attention enhances tactile sensitivity in women at risk from eating disorders

**DOI:** 10.1038/s41598-020-68500-6

**Published:** 2020-07-15

**Authors:** Sofia Sacchetti, Laura Mirams, Francis McGlone, Valentina Cazzato

**Affiliations:** School of Psychology, Liverpool John Moores University, Room 3.13, Tom Reilly Building, Byrom Street, Liverpool, L3 3AF UK

**Keywords:** Neuroscience, Psychology, Risk factors

## Abstract

We examined whether alterations in body perception in EDs extend to the integration of exteroceptive visual and tactile information. Moreover, we investigated the effect of self-focused attention on the ability to correctly detect tactile stimuli. Twenty-seven women reporting low ED symptoms, versus 26 women reporting high ED symptoms, undertook a modified version of the Somatic Signal Detection Task (SSDT), which involved detecting tactile stimuli on the cheek in the presence or absence of a concomitant light. The SSDT was completed while looking at a photograph of one’s own face, another female face, and a scrambled face. Heart rate and skin conductance were recorded continuously during the SSDT. Although ED symptoms were not associated with an overall increased tendency to misperceive touch in the presence of a light, High ED participants were differentially affected by self-focused attention. For the High ED group, physiological arousal, and tactile sensitivity (*d′*) were increased when self-focused attention was augmented. For the Low ED group, sensitivity (*d′*) and physiological arousal were higher in the control conditions. We suggest that in those with High ED symptoms, attention to the bodily self may exacerbate a predisposition to focusing on external rather than internal bodily information.

## Introduction

### Body perception and eating disorders

Eating disorders (EDs) are a family of psychopathologies characterized by a persistent disturbance of eating or eating-related behaviour, accompanied by food, weight and body-shape concerns^1^. EDs show the highest mortality rate of any mental illness^2^. However, the aetiology of these disorders remains unclear and existing treatments are limited in their effectiveness with only around 50% patients fully recovering after treatment^3,4^. In light of these data, further research is needed to foster the development of more targeted treatment programs.

It has been advocated that body misperception plays a pivotal role in the aetiology and maintenance of EDs^5,6^. Body misperception is typically expressed as (perceptual) body image distortions, i.e., visual overestimation of body size and shape that fails to reflect the true dimensions^1^. However, recent evidence suggests body misperception in EDs to be more widespread and severe than previously recognised involving different components of somatic perception.

For example, it has been found that overestimation of body size also manifest itself in the tactile modality with ED patients overestimating distances between two tactile stimuli simultaneously pressed to the skin^7,8^. Alongside, body image disturbances were also shown to extend to the body schema (i.e., the neural representation of the spatial properties of the body in action and interaction with the environment^9^) with healthy subjects reporting body image concerns, and anorectic patients underestimating the width of an aperture they could pass through, and performing the action as if their body was larger than in reality^10–12^.

Moreover, EDs have been linked to deficits in the processing and perception of internal body signals, namely interoception. Interoception can be defined as the sense of the physiological condition of the body, and includes the perception of internal organ functions, muscular and visceral stimuli, hunger, thirst, pain, and pleasure^13^. In this regard, different samples of ED patients have been found to have a reduced interoceptive sensibility, that is, a lower self-reported propensity to focus on internal bodily sensations^14–16^. These findings were partially supported also by some studies assessing objective accuracy in detecting interoceptive signals such as the heartbeat and breathing sensations, and pleasantness of touch (gentle stroking, 3 cm/s^17–21^). However, while findings on reduced interoceptive sensibility are consistent across studies, some studies failed to find a difference in interoceptive accuracy between ED patients and healthy controls possibly indicating a dissociation between self-reported and experimentally measured interoceptive abilities^22,23^.

### Multisensory integration and eating disorders

Given this previous evidence that body misperception in EDs concerns not only body image distortions but also alterations in exteroception and interoception, it has been suggested to rethink body misperception in EDs in a multisensory framework, as a general impairment in the elaboration and integration of perceptive information involving different sensory modalities. Specifically, it has been advocated that patients with EDs might have difficulty integrating visual, tactile and interoceptive information^24–26^. In this regard, informative data come from studies analysing somatosensory illusions elicited by the integration of conflicting multisensory information, such as the Rubber Hand Illusion paradigm (RHI^27^). During the RHI, watching a rubber hand being stroked synchronously with one’s own unseen hand causes the illusion of body ownership of the fake hand. The integration of contrasting tactile and visual information, indeed, induces participants to mislocate the position of their own hand as closer to the fake hand.

Although the paradigm has been found to be effective in healthy subjects, further research in ED patients has shown this population to be more inclined than controls to perceive the bodily illusion, suggesting that people with EDs may have an increased sensitivity to the visual aspects of body perception, which in turn determines alterations in multisensory integration^28,29^. Similar findings were found in regard to the Size Weight Illusion (SWI^30^). During the SWI typical individuals experience a smaller object as much heavier than a larger one having the same weight, because of an implicit assumption that weight scales with size. However, Case et al.^31^ found a sample of anorectic patients to have an atypical response to the illusion possibly due to distortions in the integration of visual and proprioceptive information.

Another paradigm that has been successfully used in assessing multisensory integration is the Somatic Signal Detection Task (SSDT^32^). In its classic version, the SSDT involves detecting near-threshold vibrations delivered on the fingertip on 50% of trials with and without a simultaneous light flashing next to the targeted finger (which also occurs on 50% of trials). The SSDT allows the investigation of how visual and tactile information are integrated, and more specifically how visual information can lead to the misperception of touch. Previous studies have shown that neurotypical subjects have a tendency to erroneously report perceiving the vibration when it was not present (i.e., make false alarms) on trials when the light flash occurs^32,33^, thus indicating that the presence of non-informative visual information can elicit a false perception in another exteroceptive modality that is touch.

The SSDT has been previously used to investigate the relation between physical and somatoform symptoms and body misperception, with participants reporting higher levels of somatoform dissociation and physical symptoms being more inclined to erroneously report touch during the SSDT as compared to controls^34,35^. However, to date the paradigm has never been tested in other clinical populations such as EDs.

Unlike different paradigms assessing multisensory integration, the SSDT takes advantage of the use of the Signal Detection Theory for analysing participants’ responses in order to obtain a thorough description of the data, with separate measures of sensitivity (*d′*, i.e., the ability to correctly discriminate whether the vibration was present or absent) and response criterion (i.e., the propensity to report feeling the vibration regardless of the type of trial^36^). Moreover, in contrast to other paradigms assessing multisensory integration, such as the RHI, during which participants are aware of a distortion in their experience of an existing touch, during the SSDT participants are unaware of whether or not their experience of touch is genuine. Therefore, it could be argued that the SSDT provides a more objective measure of exteroceptive experience. The first aim of the current study was therefore to establish whether ED symptoms are related to alterations in multisensory integration as assessed using the SSDT. To address this aim, we compared SSDT performance in two samples of participants presenting with lower vs. higher levels of subclinical ED symptoms.

Sampling a non-clinical population allows to control for possible confounding variables that can affect research in clinical samples, such as cognitive and perceptual impairments secondary to starvation, or medication treatment. Moreover, research on subclinical populations has the advantage to indicate whether somatosensory disturbances anticipate the onset of EDs, therefore giving a direction for preventive measures. As EDs are associated with body misperception and heightened propensity to experience multisensory illusions, we hypothesized that subjects with higher ED symptoms would show a stronger influence of the light on touch reports and ultimately a lower sensitivity (*d′*) in discriminating when touch was present or absent during the SSDT.

### Self-focused attention and body perception

To further understand the psychological processes affecting body misperception in EDs, we also investigated the potential influence of self-focused attention on SSDT performances. Previous studies suggest that multisensory integration and somatosensory processes can be affected by the manipulation of attention. Attention to the self (i.e., self-focused attention) can be enhanced by exposing participants to the vision of their body or their face^33,37–39,41,42^. In previous studies, vision of the body was found to enhance tactile perception in terms of decreased grating discrimination thresholds^37^, reduced two-point discrimination thresholds^38,39^, and enhanced amplitude discrimination of above-threshold stimuli^40^. Similarly, Ainley et al.^41,42^ demonstrated that vision of one’s own face can enhance interoceptive accuracy. Participants were asked to perform a heartbeat perception task (HPT) while watching a photograph of their face or their reflection in a mirror as compared to a blank screen. Results suggested that subjects were more accurate in perceiving their heart rate when self-focused attention was augmented by looking at their face.

However, less information is available about the effects of self-focused attention on somatosensory processing and multisensory integration in the context of EDs. A single study replicated Ainley et al.’^41,42^ paradigm on a sample of anorectic patients. Participants completed an HPT in two conditions: while viewing a photograph of themselves or another person^22^. In contrast with previous results on healthy participants, patients showed lower interoceptive accuracy when viewing the photograph of themselves as compared to another person. It was hypothesized that anorectic patients might find it distressing viewing their own photograph due to high levels of body dissatisfaction and might therefore be less accurate in elaborating body signals in this condition.

The second aim of our study, therefore, was to determine whether self-focused attention enhances, or is detrimental to somatosensory processing, in people with different levels of ED symptoms. This is particularly important in order to inform the design of interventions to reduce body misperception in EDs, such the body exposure and the mirror therapy, which concern manipulating attention to the bodily self to improve body satisfaction and perception^43^.

To address our aims, participants underwent a modified version of the original SSDT, during which the vibration was administered to participants’ cheeks (rather than their fingertips as in its classic version) and the light was placed in front of participants (similar to Durlik et al.^44^). The face was chosen as it represents a body part more salient for the construction of one’s own identity and body image as compared to the hand, and it was therefore more relevant for this study^45^. Participants completed the task in three experimental conditions: (1) while looking at a photograph of one’s own face for eliciting self-focused attention (Self); (2) at a photograph of another person’s face (as in Pollatos et al.^22^; Other); (3) at a scrambled face (Scrambled) as additional control condition.

Physiological arousal levels, anxiety, and attitudinal components of body image including body dysmorphic symptoms were measured in order to test whether differences in participants’ performance could be accounted for by these variables.

Following previous studies (e.g., Pollatos et al.^22^), we expected that participants presenting higher levels of ED symptoms would find the Self condition more distressing due to higher levels of anxiety, dysmorphic symptoms and body image concerns. Coherently, we expected these participants to show a stronger response in terms of physiological arousal in the Self condition, compared to participants presenting lower levels of ED symptoms. In turn, we expected this response in physiological arousal to lead to a disruption in perceptual processes and therefore to a lower sensitivity (*d′*) during the SSDT in this condition. Conversely, we expected that participants presenting lower levels of ED symptoms would show the classic self-focused attention effect, with no signs of physiological distress and higher *d′* during the Self condition.

Lastly, participants completed an HPT to assess the relationship between SSDT performance and interoceptive accuracy. A previous study by Durlik et al.^44^ showed that participants exhibiting lower interoceptive accuracy were also more inclined to misperceive touch during the SSDT as indicated by higher false alarm rates. As body misperception in EDs has been found to encompass interoception, we expected worse performance during the SSDT for high ED participants to be coupled with poorer interoceptive accuracy.

## Material and methods

Participants attended one single testing session during which they performed the SSDT in the three experimental conditions: Self, Other and Scrambled. Thus, a mixed-design was employed with Condition and Light during the SSDT (light present/light absent) as within-subjects variables, Group (High vs. Low ED) as a between subjects variable, and hit rate (HR), false alarm rate (FA), *d′* and c as dependent variables.

### Participants

Fifty-nine female participants were initially recruited from the staff and student population at Liverpool John Moores University (LJMU) via flyers and advertisements placed around the campus, and from the general population via an LJMU database of people from the general public interested in taking part in research studies. The sample size was based on a power analysis using G*Power 3.0.10^46^, which indicated that a minimum total sample of 44 participants was needed to detect a medium effect (*f* = .25) with 95% power, using a mixed design ANOVA with alpha at .05 (two tailed). However, given that it is deemed good practice to exceed the minimum number of participants indicated by an a priori power analysis, the sample size was increased to 59 participants^46,47^.

However, 4 participants were excluded from analysis because a consistent tactile threshold level during the SSDT could not be achieved [(HR) > 90% or < 10% during at least one condition of the SSDT and across Light and No Light trials]. Furthermore, another 2 subjects were excluded for having outlying scores (> 2 *SD*) in all SSDT outcome measures. Therefore, we report data from 53 subjects between 18 and 39 years of age (*M* = 23.94; *SD* = 4.90). Only females were recruited due to the fact that literature on EDs in males is still scarce, and EDs have been shown to differ significantly in terms of prevalence and phenomenological manifestations between men and women^48^.

In accordance with previous research using the SSDT, participants were right-handed (as assessed using the Edinburgh Handedness Inventory^49^), with no history or present diagnosis of any psychiatric disorder, with no impairments in tactile perception of the cheek, with no uncorrectable vision problems and not being pregnant.

Participants were preselected based on their scores on the ED Risk Composite of the Eating Disorder Inventory-3 (EDI-3)^50^. See the Materials and methods section), which is an index of the risk of developing an ED. Specifically, we preselected subjects whose scores fell into the first (below Q1 = 19) and last quartile (above Q3 = 30) of the normative distribution in the general population^51^. Participants who scored 19 or below were deemed to be at very low risk for the development of an ED and formed the Low ED group (n = 27; Age: *M* = 24.70; *SD* = 5.31). Conversely, participants who scored 30 or above were at a higher risk for EDs and were included in the High ED group (n = 26; Age: *M* = 23.15; *SD* = 4.38).

The study was carried out in accordance with the Helsinki declaration of ethical standards. The study protocol was approved by the LJMU’s University Research Ethics Committee (UREC) 18/NSP/059. All participants gave their written informed consent to take part in the study. Participants were naïve as to the true purpose of the study and were debriefed by the experimenter at the end of the testing session. Participation was rewarded with a £5 shopping voucher or course credits.

### Material and measures

#### The somatic signal detection task (SSDT)—face version^32^

Participants sat in a light attenuated room approximately 40 cm in front of a computer monitor (5:4 ratio; 270 mm × 330 mm). A tactor delivering vibrations (Z-Voom phones type YVE-01B-03, Yeil Electronics, South Korea; 1.8 cm diameter) was fixed to participants’ left cheeks using double sided adhesive tape and a bandage tape to prevent movements. Tactile stimuli (20 ms, 100 Hz vibrations) were produced by sending amplified sound files (.wav files, sine wave), controlled by E-Prime software (Psychology Software Tools Inc., Pittsburgh, PA, USA), to the tactor.

**Figure 1:**
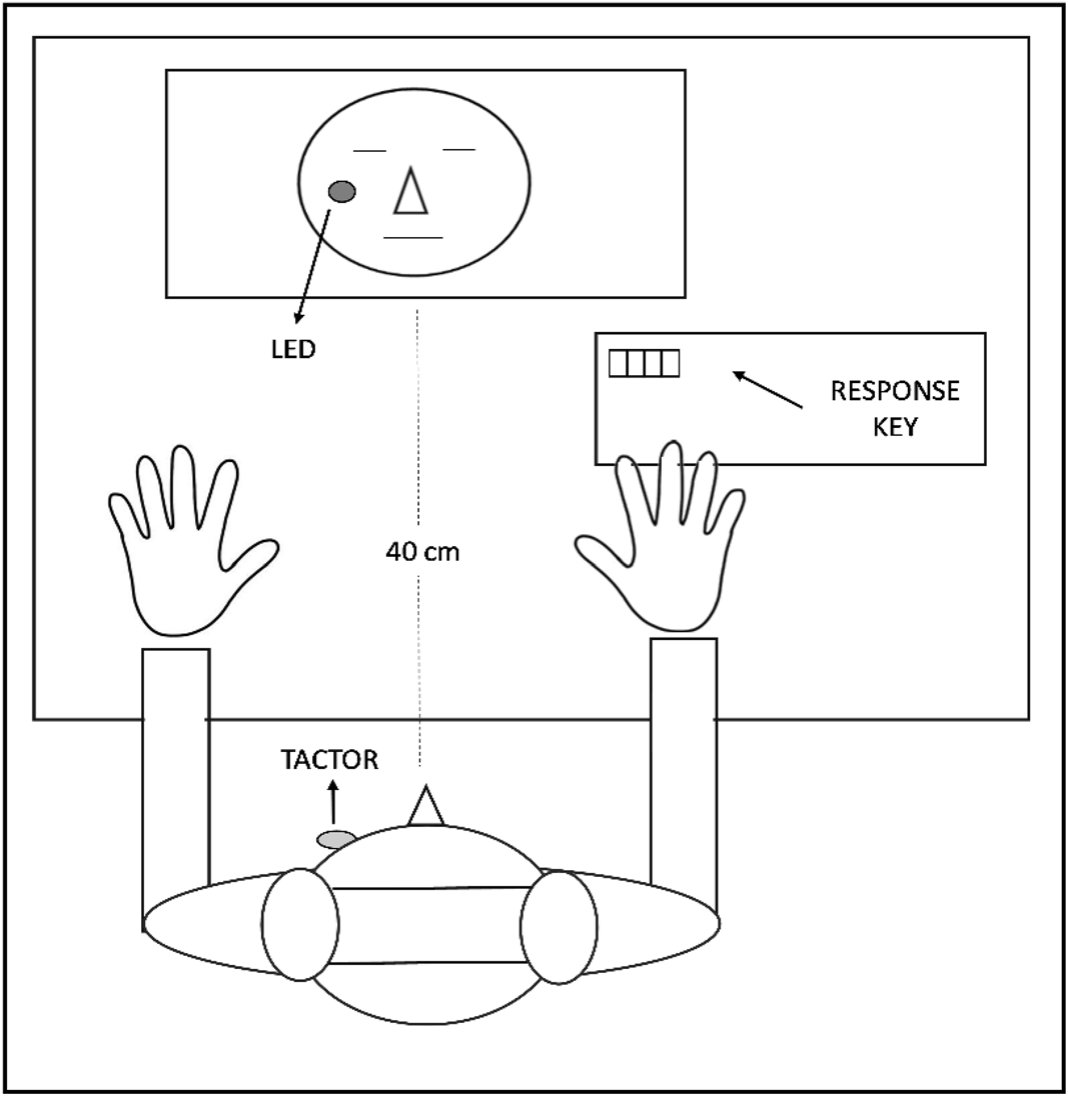
Schematic depiction of the experimental set-up for the Self/Other conditions during the SSDT.

In the Self condition, a mirror-reversed photograph of the participant’s face was presented on the computer screen during trials to induce self-focused attention. In the Other condition, a photograph of another person was presented, and in the Scrambled condition, a scrambled version of the photograph of the participant’s own face was presented (see the Procedure section for more details). The face displayed in the Other condition matched participants’ age and gender and was selected from the Chicago Face Database for average attractiveness^52^. Photographs were 768 × 583 pixels in size. During the experimental phase, a 4 mm red light emitting diode (LED) was fixed to the computer monitor mirroring the location of the tactor on the participant’s face (see Fig. 1). Therefore, during the Self and the Other conditions, the LED location corresponded to the cheek of the face depicted on the monitor. A similar version of the SSDT, in which the tactile stimulus was presented to the face, and the light was presented approximately 100 cm in front of participants, was used previously by Durlik et al.^44^, who found significant effects of the light on sensitivity and response criterion.

Gaze direction, and distance from the computer monitor during the task was controlled using a chin-rest that discouraged participants from moving their head. Throughout the experiment, participants listened to white noise via headphones to mask any informative sounds from the tactor. Underneath the headphones, participants wore a second pair of small earphones which administered auditory cues for signalling the beginning of each trial of the SSDT. The experimental set-up of the SSDT is illustrated in Fig. 1.

To control for possible confounding variables, at the end of the SSDT, participants were asked to rate on 15 cm Visual Analogue Scales (VASs) the age and attractiveness of the person shown in the Other condition. Alongside, 15 cm VASs were used to assess whether subjects interpreted the photographs presented in the Self and Other conditions assuming a first or third-person perspective. In other words, whether they interpreted themselves as active observers of the photographs or whether they perceived themselves as being watched by an external observer. No differences between groups were found in any VAS. Material and measures and results are presented in the Supplementary materials section.

#### Physiological arousal measures

Electrodermal Activity (EDA) and Electrocardiogram (ECG) signals during the SSDT were recorded using Biopac (MP150) Systems (Version 4.2, Biopac Systems Inc., CA, USA). For each condition of the SSDT, data were recorded at baseline for 5 min before the beginning of the task, and continuously throughout the whole task. ECG data were registered via a set of three electrodes applied on the right and left shoulders and on the left hip (Einthoven’s triangle). EDA data were registered via an additional set of two electrodes applied on the middle finger and index of the left hand. Both sets of electrodes were connected to the Biopac (MP150) Systems and the Biopac Student Lab Pro 3.7 software. The software was programmed to filter in real time ECG data with a band-pass of .5–35 Hz and EDA data with a band-pass of 0–35 Hz. The sampling rate for data acquisition was set at 1,000 Hz.

EDA and ECG data were then used for calculating two well-known physiological measures of arousal: Skin Conductance Levels (SCLs) and High Frequency Heart Rate Variability (HF HRV). SCLs are a measure of background tonic EDA and represent an index of the activity of the sympathetic nervous system (SNS^53,54^). The SNS is activated in response to stress and emotional stimuli. In turn, SNS activation modulates the electrical activity of the skin resulting in increased sweating and therefore increased SCLs^55^. Conversely, HF HRV (HRV in the range of 0.15–0.4 Hz) represents an index of the activity of the parasympathetic nervous system (PSNS^56^) which controls functions of the body at rest. Resting states are associated with an increased activity of the PSNS and with a corresponding increase in HF HRV. Therefore, higher arousal can be associated either with higher SCLs, or with lower HF HRV, or with a combination of the two^57^.

In the current study, changes in arousal were found to be driven by modifications in the SNS but not the PSNS. Indeed participants reported changes in SCLs across the different experimental conditions but fairly stable levels of HF HRV. HF HRV data extrapolation and results are therefore reported in the Supplementary materials section.

#### The heartbeat perception task (HPT)^58^

Interoceptive accuracy (IAcc) was assessed using the mental tracking method HPT^58^. Participants were instructed to mentally count their heartbeats only by concentrating on their body but without taking the pulse in 4 signalled time intervals ranging between 20 and 55 s. The duration of the sum of the intervals was set at 150 s for each participant. Participants were not aware of the duration of time intervals, and had no access to any indication of time (e.g. no computer or wrist watch). At the end of each interval, participants were asked to verbally report the number of heartbeats counted in that time window starting from 0 in case no heartbeats were felt. They were specifically encouraged not to use any prior knowledge about their heart rate when performing the task. Simultaneously, the actual number of heart beats occurring in each interval was recorded through a sensor fitted to the participants’ fingertips and connected to the physiological data unit Biopac (MP150) Systems. IAcc was then calculated relating the reported number of beats counted with the actual number of beats recorded (see below for data processing^42^).

### Self-report questionnaires

#### Dysmorphic concern questionnaire (DCQ)^59^

The DCQ is a 7-item self-report investigating participants’ concern about their physical appearance. Items cover topics such as the belief of being misshapen or malformed despite others’ opinion; belief in bodily malfunction (e.g. malodour); consultation with cosmetic specialists; spending excessive time worrying about appearance; and spending a lot of time covering up perceived defects in appearance. Participants were asked to rate each item on a Likert scale from a minimum of 0 (“not at all”) to a maximum of 4 (“much more than most people”). Total scores range from 0 to 28 with a critical value of 9 indicating clinical concern^60^. The DCQ was found to have a good internal consistency with α = .80^61^. The scale was administered to investigate whether Low and High ED participants differ in their levels of physical appearance concern, therefore suggesting a different interpretation of the photograph presented in the Self condition.

#### State‐trait anxiety inventory (STAI)^62^

The STAI is a 40-item self-report questionnaire for assessing anxiety. The measure includes two subscales of 20 items differentiating between State-Anxiety and Trait-Anxiety. The State-Anxiety scale assesses the intensity of anxious feelings perceived “at this moment” while the Trait-Anxiety scale assesses the tendency to experience worry and anxiety “in general”. The STAI includes items such as “I feel calm”, “I am worried” and “I feel nervous”. Items are rated on a 4-point Likert Scale ranging from “not at all” to “very much so”. Total scores range from a minimum of 20 and a maximum of 80. The STAI has been found to have an excellent internal consistency for both scales: State-Anxiety and Trait-Anxiety (α = between .89 and .92^63,64^). Scores were used to analyse whether state or trait anxiety differed in the Low versus the High ED group, possibly suggesting a different emotional response (State-Anxiety) due to the experimental manipulation.

#### Eating disorder inventory 3 (EDI‐3)^50^

The EDI‐3 is a self‐report questionnaire for the assessment of EDs. The instrument comprises 91 items organized into 12 primary scales. Three of these scales focus on ED core symptoms: Drive for Thinness, Bulimia and Body Dissatisfaction. The sum of their scores constitutes an index of the risk to develop an ED: the ED Risk Composite. The remaining 9 subscales measure general psychological functioning and other personality traits that are often related to ED symptoms: Low Self-esteem, Personal Alienation, Interpersonal Insecurity, Interpersonal Alienation, Interoceptive Deficit, Emotional Dysregulation, Perfectionism, Ascetism and Maturity Fear. The EDI-3 was administered prior to the testing session, and the ED Risk Composite was used for selecting eligible participants as explained above. Participants were asked to rate to which extent they considered each item descriptive of themselves on a 6‐point Likert scale ranging from “never” to ”always”. The EDI-3 includes items such as “I eat sweets and carbohydrates without feeling nervous”, “I think about dieting” and “I eat when I’m upset”. The EDI-3 was validated in clinical and non‐clinical samples across different cultures, and it has been found to have a good internal consistency (α = between .75 and .92 for each subscale), excellent sensitivity and specificity as well as good discriminative validity^51^.

### Design and procedure

At the beginning of each testing session, the experimenter took a photograph of the participant’s face. Photographs were taken using a Nikon D50 digital SLR camera, with a flash. Participants were standing against a grey background in a windowless testing cubicle, and they were photographed with a neutral facial expression, without hair covering their face. Each participants’ original photograph was flipped horizontally to recreate the effect of a mirror. In case the face was not perfectly centred, photographs were adjusted and cropped using Microsoft Picture Manager 2013. The mirrored photograph of the participant then was used during the Self condition of the SSDT. Subsequently, a 70 × 70 pixel scrambled version of the Self photograph was created using Matlab 9.5. The picture was then used in the Scrambled condition of the SSDT. Electrodes for recording physiological measures (EDA and ECG) were placed on participants as previously explained.

After that, participants were administered the SSDT protocol, which consisted of a thresholding procedure and an experimental phase. The SSDT protocol was repeated three times, once in each experimental condition: Self, Other and Scrambled. Photographs were displayed during both the thresholding procedure and the experimental phase. Each participant underwent all conditions in a repeated measure fashion, and the order of conditions was randomized between participants and counterbalanced. Two breaks of 5 min were administered between conditions. Throughout the SSDT, EDA and ECG data were recorded.

After completing the SSDT, participants were asked to perform the HPT, and to complete the VAS, the EHI, the STAI and the DCQ. Lastly, height and weight were measured with a stadiometer and a scale for calculating the Body Mass Index (BMI; kg/m^2^) according to the NHS online calculator. The testing procedure lasted approximately 90 min per participant.

#### SSDT

##### Thresholding procedure

Replicating the methods used by Mirams et al.^33^, before the beginning of the testing phase, participants completed a thresholding procedure to individually calibrate the strength (amplitude) of vibration. A threshold was found for each participant using a staircase procedure^65^ in which participants were presented with blocks of 13 trials: 10 tactile present (Touch) and 3 tactile absent (No Touch) trials. The beginning of each trial was signalled by a 250 ms beep sound administered through a pair of earphones. This was followed by a 1020 ms stimulus window. In Touch trails, a 20 ms tactile pulse was administered in the middle of the stimulus window (preceded and followed by a 500 ms interval). In No Touch trials, an empty 1020 ms period occurred. A prompt was then displayed instructing participants to report whether they had felt the vibration (“Yes”) or not (“No”) by pressing the corresponding keyboard keys Y and N. The instructions appeared on the top section of the computer screen in order not to hide the photograph displayed according to the experimental condition. The vibration was initially presented at the same intensity to all participants. At the end of each block, the strength of vibration was decreased if the stimulation was perceived on more than 60% of Touch trials. If the vibration was perceived on less than 40% of Touch trials, the intensity was increased. This procedure was repeated until the intensity of vibration reached the participant’s 50% threshold, defined as the intensity necessary for participants to perceive the vibration on 40–60% of Touch trials. Participants had to score within this range for two consecutive blocks at the same stimulus intensity^33^. The thresholding procedure took approximately 15 min. Immediately after completing the thresholding procedure, participants started the experimental phase of the SSDT.

##### SSDT experimental phase

Before the beginning of the experimental block, the experimenter placed the LED on the computer monitor. The experimental block of the SSDT used a repeated-measures design with tactile vibration (Touch, No Touch) and light (Light, No Light) as within-subjects variables. The tactile vibration was set at the strength individually calibrated during the thresholding procedure. Therefore, participants were administered with four different trial types: touch only (No Light/Touch); light and touch (Light/Touch); light only (Light/No Touch); and catch (No Light/No Touch)^32,33^. Each of the four trial types was repeated 10 times per block in a random order, with each block consisting of a total number of 40 trials. As for the thresholding procedure, the beginning of each trial was signalled by an auditory cue, followed by a 1020 ms stimulus window. In accordance with the original paradigm^32,33^, in touch only trials, a 20 ms tactile vibration was administered alone in the middle of the stimulus window (preceded and followed by a 500 ms interval). In light and touch trials, the LED flashed for 20 ms synchronously with the tactile vibration. In light only trials, the LED flashed for 20 ms alone. In catch trials, no stimulation was administered. At the end of each trial, participants were asked to report whether or not they felt a vibration. They were instructed to press the keyboard buttons ‘1’ for ‘definitely yes’, ‘2’ for ‘maybe yes’, ‘3’ for ‘maybe no’, or ‘4’ for ‘definitely no’^33^. Instructions appeared on the top section of the computer screen. For the purposes of this study, ‘definitely’ and ‘maybe’ responses were combined in a yes/no binary coding.

### Statistical analysis and statistical software

All Statistical analysis were performed using Statistica 8.0 (StatSoft Inc, Tulsa, Oklahoma software). All data are reported as Mean (M) and Standard Deviation (SD). A significance threshold of *p* < .05 was set for all effects. Effect sizes were estimated using partial eta square (*η*^2^) and Cohen’s d.

A series of t tests were conducted to investigate whether there were differences in age, BMI, Handedness, IAcc, DCQ, STAI, EDI-3 subscales, and VASs between the High and Low ED groups.

To determine whether the two ED groups differed in their SSDT performance, four mixed design ANOVAs were then performed using Group (High vs. Low ED) as the between-subject factor, Light (Light/No Light) and Condition (Self/Other/Scrambled) as within-subject factors, and HR, FA, d*′* and c as dependent variables. Duncan post-hoc comparisons were performed to follow-up significant interactions.

To determine whether the two ED groups differed in arousal in each condition of the SSDT, we performed a mixed-design ANOVA with Group as the between-subject factor, Condition as the within-subject factor, and SCLs as dependent variable.

For HR, d*′* and SCLs, significant results were followed-up also with t tests comparing change scores between the different experimental conditions.

Before performing the ANOVAs, all dependent variables were tested for normality, homogeneity of variance and sphericity assumptions. Whilst, HR, d*′* and c were found to be normally disturbed for all experimental conditions, FA and SCLs data were found to be not normally distributed in some experimental conditions. The data remained not normal after attempting for different transformations. However, given that the F test is fairly robust against violation of assumptions^46,47^, parametric tests were performed for FA and SCLs in coherency with the other outcome variables. (Please note that significant findings in FA and SCLs were tested also using non-parametric tests. Because results were substantially the same when using parametric tests, we then decided to report the results of parametric tests for the sake of consistency with other results.)

After controlling that the assumptions of bivariate normality and linearity were met, Pearson’s *r* partial correlations were used to further investigate relationships between variables.

## Results

### Data processing

#### SSDT scores

Responses on the SSDT were classified as hits (reports of feeling the touch on Touch trials), misses (reports of not feeling the touch on Touch trials), false alarms (erroneous reports of feeling the touch on No Touch trials) or correct rejections (reports of not feeling the touch on No Touch trials). According to the log-linear correction, hit rates (HR) were calculated using the formula [hits + .5/(hits + misses + 1)], and false alarm rates (FA) following the formula [false alarms + .5/(false alarms + correct rejections + 1)]^66^. Participants’ perceptual sensitivity (*d*′) [*z*(hits) − *z*(false alarms)] and tendency to report touch as present (response criterion, *c*) [− .5*zHR + *z*FA] were calculated using HR and FA^67^. Lower scores on *c* (*c* < 0) indicate a higher tendency to report touch (answer “yes”) across trials^33^.

#### HPT scores

IAcc was calculated for each participant as the mean score across the four trials using the formula {1/4 ∑[1 − (|recorded heartbeats − counted heartbeats|/recorded heartbeats)]} where higher scores indicate increased IAcc^42,58^.

#### SCLs data extrapolation

EDA data were recorded continuously during each condition of the SSDT. For each of the 3 conditions, 2 recordings were obtained: at baseline and during the experimental phase, resulting in a total of 6 recordings per participant. Each recording was visually inspected for artefacts which were manually removed using Biopac (MP150) Systems. Six SCLs per participant were extrapolated averaging across the EDA signal in each baseline and experimental recording. No differences between groups (Low vs. High ED) were found in baseline SCLs (bs SCLs) in any of the experimental conditions (bs SCLs Self: *t*(51) = − 1.32, *p* = .10, *d* = .36; bs SCLs Other: *t*(51) = − 1.61, *p* = .11, *d* = .44; bs SCLs Scrambled: *t*(51) = − 1.71, *p* = .09, *d* = .56). Three baseline-corrected SCLs were then calculated by subtracting baseline SCLs from their respective experimental SCLs. The baseline-corrected SCLs were later used for all the analyses described in the below results section.

### Preliminary analyses

Results of the t tests are presented in Table 1. The two groups were comparable in age and IAcc. A significant difference was found in BMI with the High ED group having a higher BMI compared to the Low ED group. The High ED group also had significantly higher scores on the DCQ and on the State-Anxiety subscale of the STAI, indicating that subjects with higher ED traits have also stronger dysmorphic concerns and experienced more anxious feelings on the day of testing. However, the two groups were comparable on the Trait-Anxiety subscale, indicating that High and Low ED groups experience similar levels of anxiety on a day to day basis. Interestingly, both groups exhibited Trait-Anxiety scores that were higher than normative scores in the general population, however they did not reach clinical standards^63^. The High ED group had significantly higher scores on all the subscales of the EDI-3 (Low Self Esteem, Personal Alienation, Interpersonal Insecurity, Interpersonal Alienation, Interoceptive Deficit, Emotional Dysregulation, Perfectionism and Ascetism) apart from the Maturity Fear subscale.

**Table 1:** Descriptive statistics for age, BMI, Handedness and scores on the IAcc, DCQ, STAI and EDI-3 subscales in each group, together with t-test statistics.

	Low ED	High ED	t	df	*p*	d
	M (SD)	M (SD)				
Age	24.70 (5.31)	23.15 (4.38)	1.15	51	.25	.32
BMI	21.30 (2.10)	28.02 (7.91)	− 4.26	51	.000	1.16
IAcc	.63 (.22)	.63 (.25)	− 0.05	51	.96	.01
DCQ	3.89 (2.15)	8 (3.68)	− 4.99	51	.000	1.36
State-Anxiety	29.92 (6.74)	37.35 (8.49)	− 3.45	51	.000	.97
Trait-Anxiety	50.58 (2.14)	49.38 (2.98)	1.66	51	.10	.46
Low Self-esteem	2.74 (3.59)	7.81 (5.41)	− 4.03	51	.000	1.10
Personal Alienation	2.18 (3.47)	7 (4.70)	− 4.25	51	.000	1.17
Interpers. Insecurity	4.77 (4.10)	8.11 (5.57)	− 2.48	51	.026	.68
Interpers. Alienation	4 (4.20)	7.88 (4.63)	− 3.20	51	.002	.88
Interoceptive Deficit	4.26 (4.89)	8.58 (4.42)	− 3.37	51	.001	.93
Emotional Dysreg	2.81 (3.54)	6 (4.75)	− 2.77	51	.008	.76
Perfectionism	6.44 (4.34)	9.69 (4.85)	− 2.57	51	.01	.71
Ascetism	2.26 (1.95)	6.30 (4.62)	− 4.13	51	.000	1.14
Maturity Fear	7.33 (4.04)	9.38 (4.72)	− 1.70	51	.09	.47

IAcc = Interoceptive Accuracy assessed by the HBP task. S-Anxiety and T-Anxiety are the two subscales of the STAI. Low Self-esteem, Personal Alienation, Interpersonal Insecurity, Interpersonal Alienation, Interoceptive Deficits, Emotional Dysregulation, Perfectionism, Ascetism and Maturity Fear are all subscales of the EDI-3.

### Main analyses

Descriptive statistics for HR, FA, *d*′ and *c* in each Light and Condition of the SSDT, in each group are presented in Table 2.

**Table 2:** Descriptive statistics for hit rate, false alarm rate, d*′* and c in each Face and Light condition during the SSDT, in each ED group.

		HR (%)	FA (%)	*d′*	*c*
		M (SD)	M (SD)	M (SD)	M (SD)
**Low ED**					
Self	Light	54.05 (17.56)	10.07 (11.54)	1.59 (.87)	.67 (.29)
	No light	55.67 (22.75)	6.60 (4.69)	1.79 (.86)	.70 (.36)
Other	Light	61.23 (23.24)	7.06 (5.24)	1.93 (.86)	.60 (.39)
	No light	51.74 (24.90)	6.83 (6.31)	1.65 (.88)	.78 (.45)
Scrambled	Light	61.92 (24.65)	7.99 (7.22)	1.91 (.89)	.57 (.49)
	No light	52.89 (23.61)	7.99 (7.62)	1.64 (.83)	.71 (.41)
**High ED**					
Self	Light	66.34 (21.75)	8.65 (8.90)	2.08 (.87)	.47 (.42)
	No light	57.93 (22.45)	5.77 (7.11)	1.93 (.68)	.74 (.41)
Other	Light	60.10 (24.83)	5.05 (3.86)	2 (.76)	.68 (.44)
	No light	56.25 (26.47)	5.05 (4.25)	1.95 (.76)	.74 (.50)
Scrambled	Light	57.45 (24.98)	9.13 (8.19)	1.69 (.80)	.61 (.48)
	No light	55.77 (22.58)	7.21 (5.29)	1.76 (.84)	.67 (.39)

#### Hit rate

**Figure 2:**
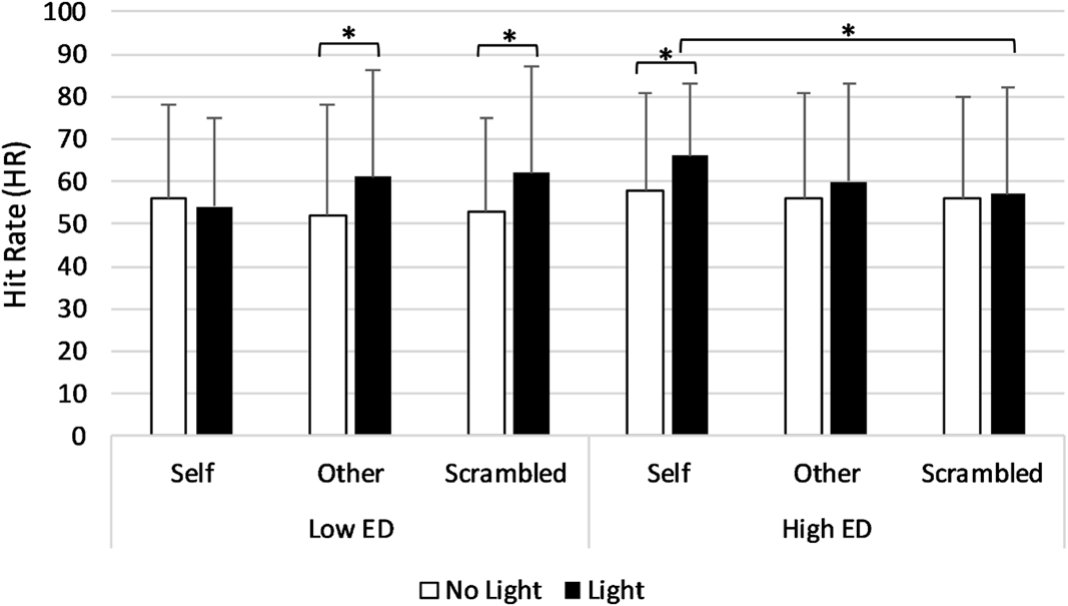
Mean HR during the SSDT in Light and No Light trials of each condition in the Low and the High ED groups. Error bars show the standard deviation. For the Low ED group, there was a significant effect of the Light in the Other (*p* = .02) and the Scrambled conditions (*p* = .02). For the High ED group, there was a significant effect of the Light in the Self condition (*p* = .03) and significantly higher hit rates in Light trials of the Self compared to the Scrambled condition (*p* = .02).

A significant main effect of Light (*F*(1,51) = 12.02, *p* = .001, *η*^2^ = .19) was found with higher HR in Light (*M* = 60.2, *SD* = 16.17) compared to No Light (*M* = 55, *SD* = 15.43) trials. Importantly, the Light × Condition × Group interaction was significant (*F*(2,102) = 3.99, *p* = .02, *η*^2^ = .07). Post-hoc comparisons showed that for the Low ED group there was a significant effect of Light in the Other condition, with higher HR in Light (*M* = 61.23, *SD* = 23.24) compared to No Light trials (*M* = 51.74, *SD* = 24.90; *p* = .02, *d* = .47). Likewise, there was a significant effect of Light in the Scrambled condition, with higher HR in Light (*M* = 61.92, *SD* = 24.65) compared to No Light trials (*M* = 52.89, *SD* = 23.61; *p* = .02, *d* = .45). However, no effect of Light was found in the Self condition (see Fig. 2).

Conversely, for the High ED group, there was a significant effect of Light only in the Self condition, with an increase in HR in Light (*M* = 66.34, *SD* = 21.75) compared to No Light trials (*M* = 57.93, *SD* = 22.45; *p* = .03, *d* = .57). However, no effects of Light were found in the Other or Scrambled conditions. Moreover, HR in Light trials of the Self condition were significantly higher than those of the Scrambled condition (*M* = 57.45, *SD* = 24.98; *p* = .02, *d* = .35). The opposite pattern was found in the Low ED group, which showed higher HR in Light trials of the Scrambled (*M* = 61.92, *SD* = 24.65) condition as opposed to the Self (*M* = 54.05, *SD* = 17.56) condition. However, this difference was found only to approach significance (*p* = .05, *d* = .27).

We further investigated these results by calculating HR change scores in Light trials from the Scrambled to the Self condition, and performed an independent sample t test to analyse group differences. Change scores showed an increase in HR from the Scrambled to the Self condition for the High ED group (*M* = 8.89, *SD* = 25.13) and a decrease for the Low ED group (*M* = − 7.87, *SD* = 29.34), and the difference in change scores between the two groups was significant (*p* = .03, *d* = .62). There were no significant main effects of Condition (*F*(2,102) = .09, *p* = .91, *η*^2^ = .002) or group (*F*(1,51) = .44, *p* = .51, *η*^2^ = .009), and no significant Condition × Group (*F*(2,102) = .62, *p* = .54, *η*^2^ = .01) or Light × Group (*F*(2,102) = .47, *p* = .62, *η*^2^ = .009) interactions.

#### False alarms

**Figure 3:**
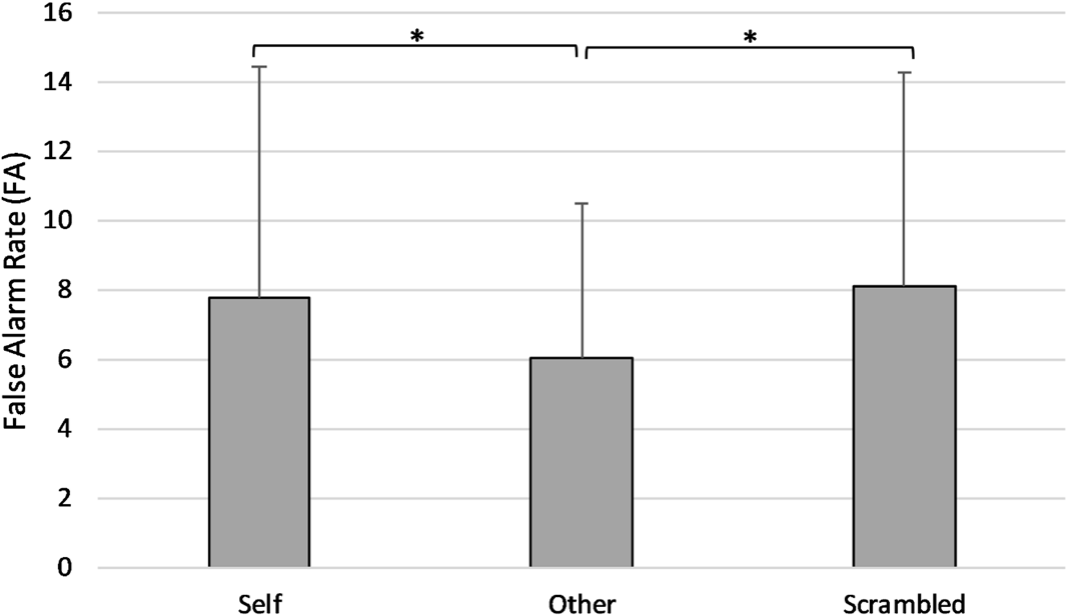
Mean FA during the SSDT in each experimental condition. Error bars show the standard deviation. FA were significantly higher in the Scrambled (*p* = .01) and the Self (*p* = .02) condition as compared to the Other condition.

There was a significant main effect of Light (*F*(1,51) = 7.30, *p* = .009, *η*^2^ = .13) with higher FA in Light (*M* = 8, *SD* = 5.96) compared to No Light trials (*M* = 6.58, *SD* = 4.38). Alongside, there was a significant main effect of Condition (*F*(1,51) = 4.30, *p* = .02, *η*^2^ = .08, see Fig. 3). FA were significantly higher in the Scrambled (*M* = 8, *SD* = .06) and Self (*M* = 8, *SD* = .07) conditions compared to the Other (*M* = 6, *SD* = .04) condition (*t*(52) = − 2.93, *p* = .005, *d* = .40; *t*(52) = − 2.34, *p* = .02, *d* = .32). Conversely, no difference in FA was found between the Self and the Scrambled conditions (*t*(52) = − 2.93, *p* = .71, *d* = .40), indicating overall a greater tendency to report false alarms in both the Scrambled and the Self-conditions compared to the Other condition. There was no significant main effect of group on FA (*F*(1,51) = .49, *p* = .49, *η*^2^ = .01), and no significant interactions between Condition × Group (*F*(2,102) = .94, *p* = .38, *η*^2^ = .02), Light × Group (*F*(1,51) = .12, *p* = .73, *η*^2^ = .002), or Condition × Light × Group (*F*(2,102) = .36, *p* = .70, *η*^2^ = .007).

#### Sensitivity (d*′*)

**Figure 4:**
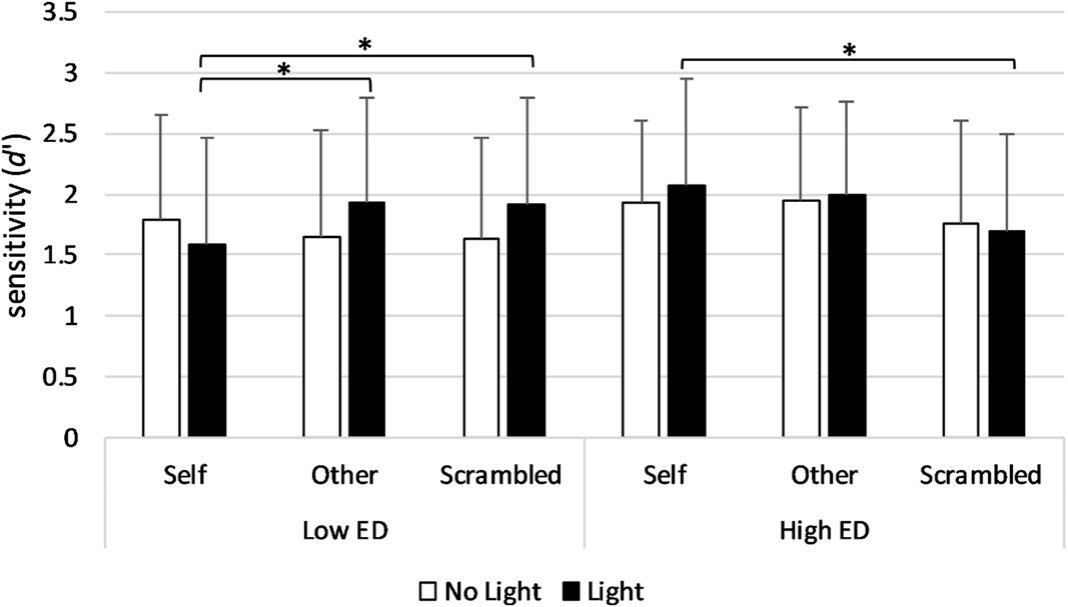
Mean *d′* during the SSDT in the Light and No Light trials of each condition in the Low and High ED groups. Error bars show the standard deviation. For the Low ED group, *d′* was significantly higher in Light trials of the Other (*p* = .039) and the Scrambled (*p* = .048) conditions as compared to the Self condition. Conversely, for the High ED group, *d′* was significantly higher in Light trials of the Self condition compared to the Scrambled condition (*p* = .02).

There was a significant Condition × Light × Group interaction (*F*(2,102) = 3.37, *p* = .04, *η*^2^ = .06). Post-hoc comparisons showed that for the Low ED group *d′* was significantly higher in Light trials of both the Other (*M* = 1.93, *SD* = .86) and the Scrambled (*M* = 1.91, *SD* = .89) conditions as compared to the Self condition (*M* = 1.59, *SD* = .87; *p* = .039, *d* = .28; *p* = .048, *d* = .28). Conversely, for the High ED group, *d′* was significantly higher in Light trials of the Self condition (*M* = 2.07, *SD* = .87) compared to the Scrambled condition (*M* = 1.69, *SD* = .80, *p* = .02, *d* = .48). Moreover, the Low ED group tended to show a lower *d′* (*M* = 1.59, *SD* = .87) in Light trials of the Self condition compared to the High ED group (*M* = 2.07, *SD* = .87; *p* = .08, *d* = .56) (see Fig. 4).

To better understand these results, we performed an independent sample t test with change scores in *d′* from the Scrambled to the Self condition during Light trials as the dependent variable. The test revealed a significant between-groups difference (*t*(51) = − 2.60, *p* = .01, *d* = .72) with an increase in *d′* from the Scrambled to the Self condition for the High ED group (*M* = .39, *SD* = .82) and a decrease for the Low ED group (*M* = − .33 *SD* = 1.16). No main effects of Condition (*F*(2,102) = .58, *p* = .56, *η*^2^ = .01), Light (*F*(1,51) = 2.10, *p* = .15, *η*^2^ = .04) or Group (*F*(1,51) = 1.04, *p* = .00, *η*^2^ = .92) were found. Alongside, the two-way interactions Condition × Group (*F*(2,102) = 1.12, *p* = .33, *η*^2^ = .02) and Light × Group (*F*(1,51) = .54, *p* = .47, *η*^2^ = .01) were not significant.

#### Response criterion (c)

The results for c mirrored the results for HR; with a significant Condition × Light × Group interaction (*F*(2,102) = 3.37, *p* = .04, *η*^2^ = .06). These results are reported in the Supplementary materials section given that they do not aid further in the interpretation of the results.

#### Skin conductance levels

**Figure 5:**
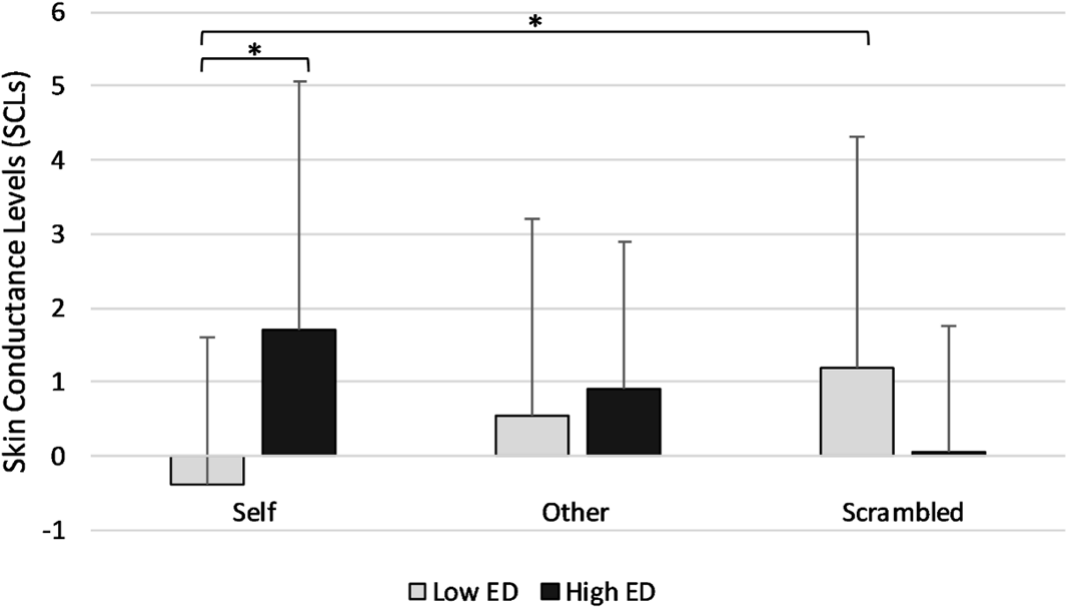
Mean SCLs in the different experimental conditions of the SSDT for the Low and the High ED groups. Error bars show the standard deviation. The High ED group showed significantly higher SCLs in the Self condition as compared to the Low ED group (*p* = .039). Moreover, for the Low ED group, SCLs in the Self condition were significantly lower compared to the Scrambled condition (*p* = .03).

Results showed a significant interaction between Group × Condition (*F*(2,102) = 5.01, *p* = .008, *η*^2^ = .09; Fig. 5). Post hoc analyses revealed a significant difference between groups in SCLs in the Self condition with the High ED group reporting significantly higher SCLs (*M* = 1.70, *SD* = 3.36) compared to the Low ED group (*M* = − .38, *SD* = 2, *p* = .009, *d* = .75). SCLs of the two groups were similar in the Other and in the Scrambled conditions (all *ps* > .10). Moreover, the Low ED group showed significantly higher SCLs in the Scrambled (*M* = 1.19, *SD* = 3.12) compared to the Self (*M* = − .38, *SD* = 2, *p* = .30, *d* = .44) condition, while the High ED group exhibited an opposite trend with higher SCLs in the Self (*M* = 1.70, *SD* = 3.36) compared to the Scrambled (*M* = .04, *SD* = 1.72, *p* = .058, *d* = .39) condition. However, this last difference was found to only approach significance. No other comparisons showed significant results (all *ps* > .10).

Lastly, we calculated change scores in SCLs between Conditions and analysed group differences using independent sample t tests. In accordance with previous results, we found the two groups to be significantly different in their change scores from the Scrambled to the Self condition (*t*(51) = − 3.01, *p* = .004, *d* = .82). While the High ED group displayed a positive change score with an increase in SCLs in the Self condition (*M* = 1.66, *SD* = 4.25), the Low ED group showed a negative change score with SCLs decreasing from the Scrambled to the Self condition (*M* = − 1.57, *SD* = 3.55, *p* = .004, *d* = .82). No significant main effect of Condition (*F*(2,102) = .18, *p* = .84, *η*^2^ = .003) or Group (*F*(1,51) = .64, *p* = .43, *η*^2^ = .012) were found in SCLs.

### Correlational analyses

#### Physiological arousal and ED symptoms in the self condition

Further correlational analyses were performed to test the hypothesis that ED symptoms in the overall sample would be associated with increased SCLs in the Self condition. Indeed, we found significant positive correlations between SCLs during the Self condition and the ED Risk Composite (*r*(53) = .36, *p* = .01) as well as the Body Dissatisfaction EDI-3 subscale (*r*(53) = .45, *p* = .001). These results suggest that participants with higher levels of ED symptoms and specifically with a higher dissatisfaction towards their body have a stronger physiological response when exposed to a photograph of themselves.

#### Physiological arousal and SSDT outcomes

Following our finding that the high ED group had higher SCLs, together with higher HR and *d*′ in the Self condition, we investigated whether higher physiological arousal would be associated with better performance during the SSDT. Therefore, we examined whether changes in HR and *d*′ from the Scrambled to the Self condition were associated with the corresponding change in SCLs. This hypothesis was partially confirmed, with a positive correlation between the change score in *d*′ from the Scrambled to the Self condition in Light trials and the corresponding change score in SCLs (*r*(53) = .30, *p* = .03), indicating that overall an increase in SCLs from the Scrambled to the Self condition was associated with a parallel increment in the ability to discriminate touch in Light trials (*d*′).

#### Interoceptive accuracy, interoceptive deficit and SSDT outcomes

Lastly, we tested whether IAcc (as measured by the HPT) and Interoceptive Deficit were associated with poorer SSDT performances. However, no significant correlations were found (all *ps* > .15).

## Discussion

The first aim of this study was to investigate the relationship between ED symptoms and multisensory integration as assessed using the SSDT. Based on previous literature linking EDs with widespread abnormalities in body perception, we expected High ED participants to be less accurate in detecting tactile stimuli during the task (show lower *d′*) as compared to the Low ED group. The second goal of the study was to investigate the effects of self-focused attention on SSDT performance. To address this issue, participants completed the SSDT in three conditions: while looking at a photograph of themselves to elicit self-focused attention (Self); while looking at a photograph of another person (Other) and whilst looking at a photograph of a scrambled face (Scrambled).

Results of the study only partially support previous research linking high ED symptoms to a greater propensity towards body misperception. In contrast with our first hypothesis, no overall differences between the Low and the High ED group were found in terms of tactile accuracy (*d′*) during the SSDT. This is not in agreement with previous findings in which ED patients were found to exhibit atypical responses to alternative paradigms assessing multisensory integration, such as the Rubber Hand Illusion (RHI) and the Size Weight Illusion (SWI^28,31^). However, it should be noted that the different paradigms here mentioned manipulate different perceptive processes. While the RHI and the SWI implicate integrating exteroceptive (visual and tactile) and interoceptive information, the SSDT assesses a different type of multisensory processes involving the integration of exclusively exteroceptive (visual and tactile) information. The discrepancy between results could therefore be better explained by the use of different instruments. More specifically, it could be the case that body misperception in EDs manifest in aberrant multisensory integration processes only when both the exteroceptive and the interoceptive modalities are implicated but not when exteroceptive information only are to be integrated.

However, we found evidence to suggest that participants with lower vs. higher ED symptoms were affected differently by our manipulation to induce self-focused attention during the SSDT. In previous research on healthy subjects, vision of one’s own face was found to induce a shift in attention toward the bodily self, ultimately enhancing perceptive accuracy^41,42^. Conversely, we proposed that vision of the face may induce an opposite decrease in perceptive accuracy in ED patients, due to the fact that ED patients may find viewing their own face highly distressing as a result of body dissatisfaction and concerns around physical appearance^22^.

Confirming our expectations, in this study the High ED group showed a higher level of distress during the Self condition of the SSDT, as indicated by a stronger physiological response (higher skin conductance levels; SCLs) in this condition. In addition, the High ED group reported greater scores on the DCQ and the State-Anxiety subscale, indicating higher levels of dysmorphic concerns and anxiety on the day of testing. Accordingly, correlational analyses on the whole sample showed that participants with higher levels of ED symptoms and specifically with a higher dissatisfaction towards their body had a stronger physiological response in the Self condition.

However, contrary to our expectations, increased physiological arousal was associated with an increase, rather than a decrease in tactile accuracy (*d′*). Indeed, the High ED group showed higher HR and *d′* during Light trials of the Self condition, compared to the Scrambled condition. Furthermore, for this group, the presence of the Light only resulted in significantly higher HR in the Self condition but did not increase HR in the control conditions.

For the Low ED group, confirming our expectation, there was no increase in physiological arousal as a response to viewing their own face. Rather, SCLs showed an opposite pattern with lower SCLs in the Self condition and higher SCLs in the control Scrambled condition. Low SCLs in the Self condition could possibly indicate a decrease in arousal and a calming effect in the presence of a highly familiar stimulus (the face of oneself) that is not experienced as distressing for those participants. Conversely, higher SCLs in the Scrambled condition may reflect that attention of Low ED participants was particularly directed towards novel stimuli as opposed to faces that are more familiar. The image of the scrambled face may therefore have been more salient for these participants due to its novelty^45,68^.

In regard to SSDT outcomes, counter to our hypothesis, for the Low ED group, self-focused attention (induced by exposure to the photograph of oneself) did not enhance perceptive accuracy. Conversely, *d′* and HR were higher in the control conditions. Indeed, Low ED participants were more accurate (*d′*) in perceiving touch in Light trials of the Scrambled as well as the Other condition compared to the Self condition. Furthermore, the occurrence of the Light was found to significantly increase HR in both the Scrambled and Other conditions but not in the Self condition.

Previous research by Pollatos et al.^22^ proposed that the anxious response experienced by high symptomatic participants in the presence of the photograph of their face may be related to poorer performances in tasks assessing body perception. This claim was formulated based on a previous study during which ED patients were found to be less interoceptively accurate during a HPT when facing a photograph of their face compared to a control condition^22^. However, results of our study show an opposite pattern with participants in the High ED group being more accurate (*d′*) during the SSDT when facing the photograph of themselves, regardless of distress experienced/increase in arousal.

In this respect, it should be noted that the two studies employed two different tasks for testing body perception tackling into different components of somatic perception: while the HPT employed by Pollatos et al.^22^ assesses interoception (the ability to perceive internal body sensations associated with heart beats), the SSDT used in this study measures the ability to perceive a near threshold, external tactile stimulus (exteroception). Therefore, it could be the case that self-focused attention has a differential effect on participants with high ED symptoms depending on the task that they are required to perform.

Furthermore, it is worth noting that body perception in EDs has been linked to a preferential reliance on visual appearance and exteroceptive information (sensory data deriving from the outer world) over perception from inside the body^69^. Accordingly, EDs have been described by the phenomenological psychology as having an “outward dispositional affective style”, which means that ED patients anchor their sense of self to a greater extent to external bodily reference points in the service of visceral and internal somatic information^70–72^. Accordingly, in the current study High ED participants were found to self-report a lower sensibility to internal body signals. However, these findings were not coupled with a lower interoceptive accuracy as measured using the HPT. The discrepancy found between interoceptive sensibility and accuracy replicates results of previous studies assessing interoception in EDs^22^. This discrepancy may be due to the fact that certain domains of interoception (i.e., the accuracy in perceiving heartbeat sensations) remain intact in EDs, or may result from the limitations of the task used. Indeed, the scientific community has recently shown growing concerns in regard to the validity of the HPT, suggesting that performance in the task may be highly influenced by non-interoceptive processes^73^.

Interestingly, an inverse relation between exteroception and interoception, similar to the one theorized in EDs, has been found also in experimental settings, where subjects with lower interoceptive awareness were found to be more sensitive to exteroceptive stimulations in the context of bodily illusions such as the RHI^74^. Consistently, it was proposed that in the absence of an accurate interoceptive representation, one’s model of the self relies predominantly on exteroception^75^.

Following this line of reasoning, it could be the case that enhancing self-focused attention in subjects with high ED symptoms exacerbates their predisposition to place more focus on exteroception rather than interoception, ultimately leading to better performances in tasks where they are required to elaborate exteroceptive information and worse performances in tasks assessing interoception. Results of this study suggested also the presence of a link between physiological arousal and participants’ variation in SSDT performance. Overall, results indicate that higher arousal was associated with a greater accuracy (*d′*) in detecting tactile information and especially in the presence of a concomitant light flashing (therefore suggesting a greater focus on visual and tactile exteroceptive information). For the Low ED group, this link was especially evident in the control Scrambled condition; while for the High ED group, that was the case in the Self condition. Accordingly, correlational analyses on the whole sample indicated that increases in SCLs from the Scrambled to the Self condition were associated with a parallel increment in the accuracy to detect touch (*d*′).

In this regard, it is worth mentioning that previous research has linked arousal to self-focused attention and perceptual accuracy. Specifically, it was proposed that self-focused attention can be induced by higher arousal (associated both with positive and negative valence) and in turn can lead to participants experiencing one’s own body as more perceptually salient^66,76,77^. Therefore, arousal could either be an alternative non-mutually exclusive explanation for participants’ greater focus on exteroceptive information or it could play a role as mediator between self-focus and perceptive processing. However, in order to better characterize how physiological arousal can influence perception, further studies will be needed assessing arousal levels during different tasks for the measurement of both interoception and exteroception, and in the presence of different type of stimuli (i.e., manipulating novelty and/or the emotional value of stimuli).

Lastly, results of this study showed that all participants reported lower FA on the SSDT in the Other condition. It could be argued the exposure to the face of another person elicited the feeling of being watched by an external observer. This, in turn, could have enhanced attention to the self (in the form of social self-focused attention), ultimately reducing touch misperception. Accordingly, a previous study investigating this area of research using the SSDT, showed that, similar to our results, participants were less inclined to report false sensations of touch in a condition intended to induce a feeling of being watched (using the presence of a video camera^44^). Possibly, these results suggest that the image of a scrambled face may be preferred as a neutral control condition instead of the photograph of another person when manipulating self-focused attention.

In conclusion, our main results demonstrate the existence of a link between ED symptoms and responses to the manipulation of self-focused attention. For subjects presenting with a higher level of ED symptoms, attention to the self is proposed to enhance the perception of exteroceptive signals. This is in line with arguments present in the previous literature that EDs are characterized by an over-investment on perceptual information coming from the outer world (exteroception) coupled with a blunted perception of bodily information coming from within the body (interoception^69–72^). Therefore, in subjects with high ED symptoms, self-focused attention is thought to exacerbate this dispositional perceptive style leading to a greater shift of attention from internal to external bodily information. Accordingly, self-focused attention may lead to better performances in tasks where subjects with high ED symptoms are required to elaborate exteroceptive information and worse performances in tasks assessing interoception.

Further research will be therefore needed to test the replicability of current findings, and the validity of the hypotheses formulated in discussing these results. Future research may benefit also from the use of eye-tracking methods to investigate differences between Low vs. High ED participants in visually inspecting photographs. Although gaze direction was minimized using a chin-rest that discouraged participants from moving their head, it is still possible that differences in SSDT performance may be due to differences in visual observation of the faces and/or LED. The design may also be implemented by the use of different familiar/unfamiliar visual stimuli, other than photographs and bodily related images to investigate specificity of results. Moreover, it should be noted that the study sampled a population of healthy subjects with different levels of ED symptoms. Although this choice allows to control for the presence of confounding variables that characterize research in clinical samples (i.e., cognitive and perceptual impairments secondary to abnormal Body Mass Index (BMI), starvation, or medication treatment), it would be worthwhile to investigate whether results of this study could be generalized to a clinical populations of ED patients.

Lastly, it is worth noticing that in this study high ED participants were also found to report a higher BMI. This is consistent with some studies that showed higher BMI levels to be predictive of future onset of an ED^78^. However, other studies failed to replicate these results by showing no influences or even an inverse relationship between BMI and ED risk^79,80^. The role of the BMI in predicting ED onset as well as its influence on perceptual processes should be therefore further investigated.

Nonetheless, the results of this study are particularly important considering that exposure to one’s own image of the body is part of different protocols for the treatment of EDs, such as the body exposure and the mirror therapy^43^. These protocols have been shown to be effective in reducing body dissatisfaction at the end of treatment^81,82^. However, the somatosensory processes involved in these protocols have not been explained up to date. The results of this study suggest a possible explanation and point out new directions for further research.

## Supplementary information

Supplementary file1 (DOCX 17 kb)

## References

[1] American Psychiatric Association (2013). Diagnostic and Statistical Manual of Mental Disorders.

[2] Arcelus J, Mitchell AJ, Wales J, Nielsen S (2011). Mortality rates in patients with anorexia nervosa and other eating disorders: A meta-analysis of 36 studies. Arch. Gen. Psychiatry.

[3] Keski-Rahkonen A (2009). Incidence and outcomes of bulimia nervosa: A nationwide population-based study. Psychol. Med..

[4] Hay P, Girosi F, Mond J (2015). Prevalence and sociodemographic correlates of DSM-5 eating disorders in the Australian population. J. Eat. Disord.

[5] Stice E (2002). Risk and maintenance factors for eating pathology: A meta-analytic review. Psychol. Bull..

[6] Levine MP, Piran N (2004). The role of body image in the prevention of eating disorders. Body Image.

[7] Keizer A, Smeets MAM, Dijkerman HC, Van den Hout M, Klugkist I, Van Elburg A, Postma A (2011). Tactile body image disturbance in anorexia nervosa. Psychiatry Res..

[8] Keizer A, Smeets MAM, Dijkerman HC, van Elburg A, Postma A (2012). Aberrant somatosensory perception in anorexia nervosa. Psychiatry Res..

[9] Berlucchi G, Aglioti SM (2010). The body in the brain revisited. Exp. Brain Res..

[10] Guardia D (2012). Imagining one’s own and someone else’s body actions: Dissociation in anorexia nervosa. PLoS ONE.

[11] Keizer A, Smeets MA, Dijkerman HC, Uzunbajakau SA, van Elburg A, Postma A (2013). Too fat to fit through the door: First evidence for disturbed body-scaled action in anorexia nervosa during locomotion. PLoS ONE.

[12] Irvine KR, McCarty K, McKenzie KJ, Pollet TV, Cornelissen KK, Tovée MJ, Cornelissen PL (2019). Distorted body image influences body schema in individuals with negative bodily attitudes. Neuropsychologia.

[13] Craig AD (2002). How do you feel? Interoception: The sense of the physiological condition of the body. Nat. Rev. Neurosci..

[14] Garfinkel SN, Seth AK, Barrett AB, Suzuki K, Critchley HD (2015). Knowing your own heart: Distinguishing interoceptive accuracy from interoceptive awareness. Biol. Psychol..

[15] Fassino S, Pierò A, Gramaglia C, Abbate-Daga G (2004). Clinical, psychopathological and personality correlates of interoceptive awareness in anorexia nervosa, bulimia nervosa and obesity. Psychopathology.

[16] Matsumoto R (2006). Regional cerebral blood flow changes associated with interoceptive awareness in the recovery process of anorexia nervosa. Prog. Neuropsychopharmacol. Biol. Psychiatry.

[17] Pollatos O (2008). Reduced perception of bodily signals in anorexia nervosa. Eat. Behav..

[18] Klabunde M, Acheson DT, Boutelle KN, Matthews SC, Kaye WH (2013). Interoceptive sensitivity deficits in women recovered from bulimia nervosa. Eat. Behav..

[19] Khalsa SS, Craske MG, Li W, Vangala S, Strober M, Feusner JD (2015). Altered interoceptive awareness in anorexia nervosa: Effects of meal anticipation, consumption and bodily arousal. Int. J. Eat. Disord..

[20] Crucianelli L, Cardi V, Treasure J, Jenkinson PM, Fotopoulou A (2016). The perception of affective touch in anorexia nervosa. Psychiatry Res..

[21] Bischoff-Grethe A, Wierenga CE, Berner LA, Simmons AN, Bailer U, Paulus MP, Kaye WH (2018). Neural hypersensitivity to pleasant touch in women remitted from anorexia nervosa. Transl. Psychiatry.

[22] Pollatos O, Georgiou E (2016). Normal interoceptive accuracy in women with bulimia nervosa. Psychiatry Res..

[23] Eshkevari E, Rieger E, Musiat P, Treasure J (2014). An investigation of interoceptive sensitivity in eating disorders using a heartbeat detection task and a self-report measure. Eur. Eat. Disord. Rev..

[24] Keizer A, van Elburg A, Helms R, Dijkerman HC (2016). A virtual reality full body illusion improves body image disturbance in anorexia nervosa. PLoS ONE.

[25] Gadsby S (2017). Distorted body representations in anorexia nervosa. Conscious. Cogn..

[26] Riva G, Gaudio S (2018). Locked to a wrong body: Eating disorders as the outcome of a primary disturbance in multisensory body integration. Conscious. Cogn..

[27] Botvinick M, Cohen J (1998). Rubber hands ‘feel’ touch that eyes see. Nature.

[28] Eshkevari E, Rieger E, Longo MR, Haggard P, Treasure J (2012). Increased plasticity of the bodily self in eating disorders. Psychol. Med..

[29] Caglar-Nazali HP (2014). A systematic review and meta-analysis of ‘systems for social processes’ in eating disorders. Neurosci. Biobehav. Rev..

[30] Charpentier A (1891). Analyse experimentale de quelques elements de la sensation de poids. Arch. Physiol. Norm. Pathol..

[31] Case LK, Wilson RC, Ramachandran VS (2012). Diminished size–weight illusion in anorexia nervosa: Evidence for visuo-proprioceptive integration deficit. Exp. Brain Res..

[32] Lloyd DM, Mason L, Brown RJ, Poliakoff E (2008). Development of a paradigm for measuring somatic disturbance in clinical populations with medically unexplained symptoms. J. Psychosom. Res..

[33] Mirams L, Poliakoff E, Brown RJ, Lloyd DM (2010). Vision of the body increases interference on the somatic signal detection task. Exp. Brain Res..

[34] Brown RJ, Brunt N, Poliakoff E, Lloyd DM (2010). Illusory touch and tactile perception in somatoform dissociators. J. Psychosom. Res..

[35] Brown RJ (2012). Physical symptom reporting is associated with a tendency to experience somatosensory distortion. Psychosom. Med..

[36] Smeets MA, Ingleby JD, Hoek HW, Panhuysen GE (1999). Body size perception in anorexia nervosa: A signal detection approach. J. Psychosom. Res..

[37] Taylor-Clarke M, Kennett S, Haggard P (2004). Persistence of visual–tactile enhancement in humans. Neurosci. Lett..

[38] Kennett S, Taylor-Clarke M, Haggard P (2001). Noninformative vision improves the spatial resolution of touch in humans. Curr. Biol..

[39] Serino A, Padiglioni S, Haggard P, Làdavas E (2009). Seeing the hand boosts feeling on the cheek. Cortex.

[40] Harris JA, Arabzadeh E, Moore CA, Clifford CW (2007). Noninformative vision causes adaptive changes in tactile sensitivity. J. Neurosci..

[41] Ainley V, Tajadura-Jiménez A, Fotopoulou A, Tsakiris M (2012). Looking into myself: Changes in interoceptive sensitivity during mirror self-observation. Psychophysiology.

[42] Ainley V, Tsakiris M (2013). Body conscious? Interoceptive awareness, measured by heartbeat perception, is negatively correlated with self-objectification. PLoS ONE.

[43] Vocks S, Wächter A, Wucherer M, Kosfelder J (2008). Look at yourself: Can body image therapy affect the cognitive and emotional response to seeing oneself in the mirror in eating disorders?. Eur. Eat. Disord. Rev..

[44] Durlik C, Cardini F, Tsakiris M (2014). Being watched: The effect of social self-focus on interoceptive and exteroceptive somatosensory perception. Conscious. Cogn..

[45] Haxby JV, Hoffman EA, Gobbini MI (2000). The distributed human neural system for face perception. Trends Cogn. Sci..

[46] Faul F, Erdfelder E, Lang AG, Buchner A (2007). G* Power 3: A flexible statistical power analysis program for the social, behavioral, and biomedical sciences. Behav. Res. Methods.

[47] Field A (2013). Discovering Statistics Using IBM SPSS Statistics.

[48] Stanford SC, Lemberg R (2012). A clinical comparison of men and women on the Eating Disorder Inventory-3 (EDI-3) and the Eating Disorder Assessment for Men (EDAM). Eat. Disord..

[49] Oldfield RC (1971). The assessment and analysis of handedness: The Edinburgh inventory. Neuropsychologia.

[50] Garner DM, Brownell KD, Fairburn CG (1995). Measurement of eating disorder psychopathology. Eating Disorders and Obesity: A Comprehensive Handbook.

[51] Clausen L, Rosenvinge JH, Friborg O, Rokkedal K (2011). Validating the Eating Disorder Inventory-3 (EDI-3): A comparison between 561 female eating disorders patients and 878 females from the general population. J. Psychopathol. Behav. Assess..

[52] Ma DS, Correll J, Wittenbrink B (2015). The Chicago face database: A free stimulus set of faces and norming data. Behav. Res. Methods.

[53] Sztajzel J (2004). Heart rate variability: A noninvasive electrocardiographic method to measure the autonomic nervous system. Swiss Med. Wkly..

[54] Mendes WB, Harmon-Jones E, Beer JS (2009). Assessing autonomic nervous system activity. Methods in Social Neuroscience.

[55] Critchley HD (2002). Electrodermal responses: What happens in the brain. Neuroscientist.

[56] Camm AJ, Malik M, Bigger JT, Breithardt G, Cerutti S, Cohen RJ, Coumel P, Fallen EL, Kennedy HL, Kleiger RE, Lombardi F, Malliani A, Moss AJ, Rottman JN, Schmidt G, Schwartz PJ, Singer DH (1996). Heart rate variability: Standards of measurement, physiological interpretation and clinical use. Task Force of the European Society of Cardiology and the North American Society of Pacing and Electrophysiology. Circulation.

[57] Berntson GG, Cacioppo JT, Quigley KS (1991). Autonomic determinism: The modes of autonomic control, the doctrine of autonomic space, and the laws of autonomic constraint. Psychol. Rev..

[58] Schandry R (1981). Heart beat perception and emotional experience. Psychophysiology.

[59] Oosthuizen P, Lambert T, Castle DJ (1998). Dysmorphic concern: Prevalence and associations with clinical variables. Aust. N. Z. J. Psychiatry.

[60] Mancuso SG, Knoesen NP, Castle DJ (2010). The Dysmorphic Concern Questionnaire: A screening measure for body dysmorphic disorder. Aust. N. Z. J. Psychiatry.

[61] Jorgensen L, Castle D, Roberts C, Groth-Marnat G (2001). A clinical validation of the Dysmorphic Concern Questionnaire. Aust. N. Z. J. Psychiatry.

[62] Spielberger CD (2010). State-Trait Anxiety Inventory. The Corsini Encyclopedia of Psychology.

[63] Bieling PJ, Antony MM, Swinson RP (1998). The State-Trait Anxiety Inventory, Trait version: Structure and content re-examined. Behav. Res. Ther..

[64] Kabacoff RI, Segal DL, Hersen M, Van Hasselt VB (1997). Psychometric properties and diagnostic utility of the Beck Anxiety Inventory and the State-Trait Anxiety Inventory with older adult psychiatric outpatients. J. Anxiety Disord..

[65] Cornsweet TN (1962). The staircrase-method in psychophysics. Am. J. Psychol..

[66] Snodgrass JG, Corwin J (1988). Pragmatics of measuring recognition memory: Applications to dementia and amnesia. J. Exp. Psychol. Gen..

[67] Macmillan NA, Creelman CD (1991). Detection Theory: A User's Guide.

[68] Wegner DM, Giuliano T (1980). Arousal-induced attention to self. J. Pers. Soc. Psychol..

[69] Mehling WE, Gopisetty V, Daubenmier J, Price CJ, Hecht FM, Stewart A (2009). Body awareness: Construct and self-report measures. PLoS ONE.

[70] Arciero G, Guidano VF, Neimeyer RA, Raskin JD (2000). Experience, explanation, and the quest for coherence. Constructions of Disorder: Meaning-Making Frameworks for Psychotherapy.

[71] Arciero G, Gaetano P, Maselli P, Gentili N (2003). Identity, personality and emotional regulation. Constr. Hum. Sci..

[72] Mazzola V, Marano G, Biganzoli EM, Boracchi P, Lanciano T, Arciero G, Bondolfi G (2014). The In–Out dispositional affective style questionnaire (IN–OUT DASQ): An exploratory factorial analysis. Front. Psychol..

[73] Desmedt O, Luminet O, Corneille O (2018). The heartbeat counting task largely involves non-interoceptive processes: Evidence from both the original and an adapted counting task. Biol. Psychol..

[74] Tsakiris M, Jiménez AT, Costantini M (2011). Just a heartbeat away from one's body: Interoceptive sensitivity predicts malleability of body-representations. Proc. R. Soc. B Biol. Sci..

[75] Fotopoulou A, Tsakiris M (2017). Mentalizing homeostasis: The social origins of interoceptive inference. Neuropsychoanalysis.

[76] Salovey P (1992). Mood-induced self-focused attention. J. Pers. Soc. Psychol..

[77] Liao CM, Masters RS (2002). Self-focused attention and performance failure under psychological stress. J. Sport Exerc. Psychol..

[78] Killen JD (1996). Weight concerns influence the development of eating disorders: A 4-year prospective study. J. Consult. Clin. Psychol..

[79] Patton GC, Selzer R, Coffey CCJB, Carlin JB, Wolfe R (1999). Onset of adolescent eating disorders: Population based cohort study over 3 years. BMJ.

[80] Stice E, Gau JM, Rohde P, Shaw H (2017). Risk factors that predict future onset of each DSM–5 eating disorder: Predictive specificity in high-risk adolescent females. J. Abnorm. Psychol..

[81] Moreno-Domínguez S, Rodríguez-Ruiz S, Fernández-Santaella MC, Jansen A, Tuschen-Caffier B (2012). Pure versus guided mirror exposure to reduce body dissatisfaction: A preliminary study with university women. Body Image.

[82] Díaz-Ferrer S, Rodríguez-Ruiz S, Ortega-Roldán B, Moreno-Domínguez S, Fernández-Santaella MC (2015). Testing the efficacy of pure versus guided mirror exposure in women with bulimia nervosa: A combination of neuroendocrine and psychological indices. J. Behav. Ther. Exp. Psychiatry.

