# Supplementary material for: Self-focused attention enhances tactile sensitivity in women at risk from eating disorders
Source: Sci Rep. 2020 Jul 15;10:11614. doi: 10.1038/s41598-020-68500-6 (PMC7363881; doi:10.1038/s41598-020-68500-6)
Supplement: Supplementary file 1 — Supplementary file1 (DOCX 17 kb) [file 41598_2020_68500_MOESM1_ESM.docx]

**6 Supplementary material**

**6.1 High Frequency Heart Rate Variability (HF HRV)**

*6.1.1 HF HRV data extrapolation*

ECG data were recorded continuously during each condition of the SSDT. For each of the 3 conditions, 2 recordings were obtained: at baseline and during the experimental phase, resulting in a total of 6 recordings per participant. Each recording was visually inspected for artefacts which were manually removed using Biopac (MP150) Systems. ECG signals were then imported into Kubios HRV software (University of Kuopio, Kuopio, Finland) for the frequency domain measure of the High Frequency band (0.15-0.4 Hz). Inter-beat (RR) intervals were retrieved from the original ECG signal and the smoothness priors method was applied to remove slow non-stationary trends from the HRV signal. Frequency domain estimates of HF HRV (in normalized units) were then derived using the power spectrum density with the fast Fourier transformation-based Welch’s periodogram method. Six estimates of HF HRV were therefore obtained for each participant (one estimate at baseline and one during the experimental phase of each of the 3 conditions of the SSDT).

Crucially, there were no differences between groups (Low vs. High ED) in baseline HF HRV (bs HF HRV) in any of the experimental conditions (bs HF HRV Self: *t*(51) = -1.85, *p* = .07, *d* = .51; bs HF HRV Other: *t*(51) = -1.90, *p* = .06, *d* = .52; bs HF HRV Scrambled: *t*(51) = -1.71, *p* = .25, *d* = .33). Three baseline-corrected estimates of HF HRV were then calculated by subtracting baseline values from their respective experimental values. The baseline-corrected estimates were later used for all the analyses described in the below results section.

*6.1.2 Results*

There were no significant main effects of Condition (*F*(2,102) = .98, *p* = .38, η^2^ = .02) or group (*F*(1,51) = .19, *p* = .66, η^2^ = .004), as well as no significant Condition × Group interaction (*F*(2,102) = .42, *p* = .65, η^2^ = .008). No significant correlations were found between HF VRT and SCLs (all *ps* > .11). These results were supported by previous research that showed the SNS and the PSNS to respond independently to environmental stimuli (Berntson, Cacioppo & Quigley, 1991).

**6.2 Visual Analogues Scales (VAS)**

*6.2.1 Methods*

Self-report 15 cm VAS were administered at the end of the SSDT to investigate how participants interpreted the photograph of another person (Other) and the photograph of themselves (Self). Specifically, participants were asked to answer on a scale from 0 (“not at all”) to 100 (“very much so”) for the following questions: “How strongly did it feel as though someone/yourself was watching you?” and “How strongly did it feel as though you were watching the other person/yourself?”. The first two questions investigated whether participants assumed a third-person/allocentric perspective, which implies perceiving oneself from the view point of an external observer (VAS Allocentric Other, VAS Allocentric Self). Conversely, the other two questions assessed whether participants assumed a first-person/egocentric perspective as active observers of external objects (VAS Egocentric Other, VAS Egocentric Self).

Two additional 15 cm VAS were added to control for the age and attractiveness of the person shown in the Other condition. Specifically, participants were asked to rate the level of attractiveness from a minimum of 0 to a maximum of 5 in accordance to the scale used by the Chicago Face Database (VAS Attractiveness), and then to estimate their age (VAS Age).

*6.2.1 Results*

No differences between the two groups were found on the perspective-taking VASs, suggesting that participants with High vs. Low ED symptoms did not differ in the way they interpreted the photographs presented during the SSDT. Both groups self-reporting to be more inclined to assume a first-person (VAS Egocentric Self, VAS Egocentric Other) rather than a third-person perspective (VAS Allocentric Self, VAS Allocentric Other) in both the Self and the Other conditions. Alongside, no between groups differences were found on the VAS Age and the VAS Attractiveness. Results of both groups were in line with the normative scores of the Chicago Face Database Results of comparisons are presented in the Table 1a.

Table 1a

*Descriptive statistics for VASs as dependent variables.*

|  | Low ED | High ED |  |  |  |  |
| --- | --- | --- | --- | --- | --- | --- |
|  | M (SD) | M (SD) | t | df | p | d |
| VAS Egocentric Self | 39.36 (28.46) | 46.58 (30.01) | -.88 | 51 | .38 | .25 |
| VAS Allocentric Self | 33.96 (28.17) | 41.15 (31.79) | -.87 | 51 | .39 | .24 |
| VAS Egocentric Other | 59.33 (26.82) | 55.11 (33.34) | .51 | 51 | .85 | .14 |
| VAS Allocentric Other | 47.30 (27.09) | 45.81 (29.47) | .19 | 51 | .38 | .05 |
| VAS Attractiveness | 3.06 (.90) | 2.64 (.94) | 1.65 | 51 | .10 | .46 |
| VAS age | 25.59 (3.56) | 25.96 (4) | -.35 | 51 | .72 | .10 |

**6.3 SSDT results for response criterion (c)**

There was a main effect of Light (*F*(1,51) = 19.57, *p* = .000, η^2^ = .28) with a lower *c* in Light (*M* = .60, *SD* = .29), compared to No Light (*M* = .72, *SD* = .30) trials, indicating an overall greater tendency to report touch when the Light was present. This was corroborated by a significant interaction of Condition × Light × Group (*F*(2,102) = 3.37, *p* = .04, η^2^ = .06). Post-hoc comparisons showed that for the Low ED group, there was an effect of the Light in the Other condition, with a lower *c* in Light (*M* = .67, *SD* = .29), compared to No Light trials (*M* = .78, *SD* = .45, *p* = .04, *d* = .35). Conversely, for the High ED group, there was an effect of the Light in the Self condition, with a lower *c* in Light (*M* = .47, *SD* = .42), compared to No Light trials (*M* = .74, *SD* = .41, *p* = .002, *d* = .87). Alongside, for the High ED group, *c* in Light trials was found to be lower in the Self (*M* = .47, *SD* = .42) condition compared to the Other condition (*M* = .68, *SD* = .44, *p* = .01, *d* = .37). No main effects of Condition (*F*(2,102) = .64, *p* = .53, η^2^ = .01) or Group (*F*(1,51) = 1.04, *p* = .00, η^2^ = .92) were found. Alongside, the two-way interactions Condition × Group (*F*(2,102) = 1.12, *p* = .33, η^2^ = .02) and Light × Group (*F*(1,51) = .54, *p* = .47, η^2^ = .01) were not significant.
